# Bee Trypanosomatids: First Steps in the Analysis of the Genetic Variation and Population Structure of *Lotmaria passim*, *Crithidia bombi* and *Crithidia mellificae*

**DOI:** 10.1007/s00248-021-01882-w

**Published:** 2021-10-05

**Authors:** Carolina Bartolomé, María Buendía-Abad, Concepción Ornosa, Pilar De la Rúa, Raquel Martín-Hernández, Mariano Higes, Xulio Maside

**Affiliations:** 1 Grupo de Medicina Xenómica, CIMUS, Universidade de Santiago de Compostela, 15782 Santiago de Compostela, Galicia Spain; 2 Instituto de Investigación Sanitaria de Santiago (IDIS), 15706 Santiago de Compostela, Galicia Spain; 3 Instituto Regional de Investigación y Desarrollo Agroalimentario y Forestal (IRIAF), Laboratorio de Patología Apícola, Centro de Investigación Apícola y Agroambiental (CIAPA), Consejería de Agricultura de la Junta de Comunidades de Castilla-La Mancha, 19180 Marchamalo, Spain; 4 Departamento de Biodiversidad, Ecología y Evolución, Facultad de Ciencias Biológicas, Universidad Complutense de Madrid, 28040 Madrid, Spain; 5 Departamento de Zoología y Antropología Física, Facultad de Veterinaria, Universidad de Murcia, 30100 Murcia, Spain; 6 Instituto de Recursos Humanos para la Ciencia y la Tecnología, Fundación Parque Científico Tecnológico de Albacete, 02006 Albacete, Spain

**Keywords:** Population genetics, Genetic diversity, Population structure, *Lotmaria passim*, *Crithidia mellificae*, *Crithidia bombi*

## Abstract

Trypanosomatids are among the most prevalent parasites in bees but, despite the fact that their impact on the colonies can be quite important and that their infectivity may potentially depend on their genotypes, little is known about the population diversity of these pathogens. Here we cloned and sequenced three non-repetitive single copy *loci* (*DNA topoisomerase II*, *glyceraldehyde-3-phosphate dehydrogenase* and *RNA polymerase II large subunit*, *RPB1*) to produce new genetic data from *Crithidia bombi*, *C. mellificae* and *Lotmaria passim* isolated from honeybees and bumblebees. These were analysed by applying population genetic tools in order to quantify and compare their variability within and between species, and to obtain information on their demography and population structure. The general pattern for the three species was that (1) they were subject to the action of purifying selection on nonsynonymous variants, (2) the levels of within species diversity were similar irrespective of the host, (3) there was evidence of recombination among haplotypes and (4) they showed no haplotype structuring according to the host. *C. bombi* exhibited the lowest levels of synonymous variation (*π*_*S*_= 0.06 ± 0.04 %) — and a mutation frequency distribution compatible with a population expansion after a bottleneck — that contrasted with the extensive polymorphism displayed by *C. mellificae* (*π*_*S*_= 2.24 ± 1.00 %), which likely has a more ancient origin. *L. passim* showed intermediate values (*π*_*S*_= 0.40 ± 0.28 %) and an excess of variants a low frequencies probably linked to the spread of this species to new geographical areas.

**Supplementary Information:**

The online version contains supplementary material available at 10.1007/s00248-021-01882-w.

## Introduction

Trypanosomatids (Protozoa: Trypanosomatida: Trypanosomatidae) are unicellular flagellates that parasitise a wide variety of organisms [1, 2]. According to their life cycle, they can be divided into monoxenous (with a single host) and dixenous (with two hosts). The vast majority of monoxenous trypanosomatids, which are the predominant type, are restricted to insects, especially Diptera and Heteroptera but also Siphonaptera, Blattodea, Mecoptera, Lepidoptera and Hymenoptera [3, 4].

Despite the reduced number of trypanosomatid species reported in bees (all of them included within the subfamily Leishmaniinae) — *Crithidia mellificae* Langridge and McGhee, 1967, *Crithidia bombi* Lipa and Triggiani, 1988, *Crithidia expoeki* Schmid-Hempel and Tognazzo, 2010, *Lotmaria passim* Evans and Schwarz, 2014, and a few others [5, 6] — their prevalence [6–9] and impact on the host [10–13] can be quite important.

*C. mellificae* and *L. passim* infect predominantly honeybees, although both species have been found in other Hymenoptera [8, 14–17]; the same holds for *C. bombi* which, regardless of being a common parasite of bumblebees (as is *C. expoeki*), has been occasionally detected in *Apis mellifera* Linnaeus, 1758 [6, 14, 18]. The identification of these pathogens in species sharing the same environment highlights the importance of spillover events in their circulation among arthropod communities (reviewed by [19]). Considering that parasites may adapt to infect new hosts (with the concomitant risk that this represents for the ecosystems), and that the success of such infections has been associated with variation among genotypes [20], it is surprising how little is known about the diversity and the population genetics of bee trypanosomatids.

So far, most studies addressing these matters have been performed in *C. bombi* using microsatellite markers [21, 22]. These exhibit repetitive sequences that usually present a much higher mutation rate than the rest of the genome, which may interfere not only with the assessment of the levels of diversity of a species but also with that of other population genetics estimates [23, 24]. Another approach used to analyse the variability of bee trypanosomatids is the sequencing of other nuclear *loci*, either individually [8, 25, 26] or at the genomic level [27]. Although both approximations provided interesting insights into the amount of genetic variation and/or gene copy number, only a few of these studies used population genetic statistics to quantify and compare these parameters within and between species. The use of these tools is essential to interpret molecular data and to determine the genetic bases for the adaptation to the environment, such as revealing the influence of different evolutionary forces on the amount of variation detected or uncover the existence of genetic differentiation among parasite populations from different geographical regions or hosts. This in turn allows not only to evaluate potential changes in the structure and distribution of a species (that can help to develop better conservation strategies) but also to infer their demographic history, providing extremely valuable information for applied research in many different fields (ecology, environmental sciences, wildlife conservation or biogeography, among others) [28–30].

## Materials and Methods

### Samples and DNA Extraction

Seven *A. mellifera* worker bees and five *Bombus terrestris* (Linnaeus, 1758) field samples from different areas of Spain (Supplemental Fig. 1), identified as trypanosomatid-positive by PCR [31], were selected for this study. Direct sequencing of their amplicons revealed the presence of *L. passim* in five of them (PA11-831, PA11-847, PA11-853, ITS2 PR13-21 and ITS3 PR13-21), *C. mellificae* in two (PA14-0015 and PA14-0044) and *C. bombi* in all *Bombus* specimens (B14.213, 14_349, 14_351, 14_373 and 14_395). To increase the size of the *C. mellificae* dataset, we also included the reference strain ATCC 30254. This was first grown in ATCC medium 355 and later sub-cultivated in Brain Heart Infusion (BHI) medium. Individual colonies were re-suspended in milliQ water (PCR-quality) and processed for DNA extraction.

DNA extractions from *A. mellifera* and the ATCC 30254 strain were carried out as described in Cepero et al*.* [25], using the BS96 DNA Tissue extraction protocol on a BioSprint workstation (Qiagen), whereas those from *B. terrestris* were performed using a modification of the Chelex protocol [32].

### Primer Design

The amino acid sequences of three *loci* (*DNA topoisomerase II*, *TOPII*; *glyceraldehyde-3-phosphate dehydrogenase*, *GAPDH*; and *RNA polymerase II large subunit*, *RPB1*) were obtained from the draft genome of *L. passim* SF. These were used to conduct TBLASTN searches for orthologs in other genomes of the Trypanosomatidae family. Their outputs were aligned using Bioedit [33] and used to design broad-range primers with primer BLAST (https://www.ncbi.nlm.nih.gov/tools/primer-blast/). The selected oligonucleotides were located within the most conserved regions of the markers and their sequences were as follows: Tryp-1F (CCGAGTACTTCKCSTACCAG) and Tryp-1R (AGCCGAGGATGCCCTTCAT) [25] for *GAPDH*, TrypRPB1-F (AGGCGGAGCTGATYGAGATG) and TrypRPB1-R (ACCGAGAAGGCRAAGCARTAG) for *RPB1* and TrypTOP-F (CACAAGCGCATYATGGACCT) and TrypTOP-R (TTRCTCTGCGAGTCGAACTT) for *TOPII*, respectively.

In several samples, these primers amplified simultaneously more than one trypanosomatid species (see ‘Results’ section), so, in order to amplify them separately, new sets of species-specific primers were designed (Supplemental Table 1).

### PCR, Cloning and Sequencing

PCR reactions were performed using the Phusion High-Fidelity DNA Polymerase (Thermo Scientific) in 20-μl volumes containing 9.4 μl of H_2_O, 4 μl of 5X Phusion HF Buffer, 0.4 μl of dNTP mix 10 mM, 2 μl of each primer 5 μM, 0.2 μl of Phusion DNA Polymerase and 2 μl of DNA. Cycling conditions consisted of an initial denaturalization at 98 °C for 30 s, followed by 35 cycles of denaturalization at 98 °C for 10 s, primer annealing at 62°C (for *TOPII* and *GAPDH*) or 65 °C (for *RPB1*) for 30 s (the Phusion DNA polymerase requires higher annealing temperatures than Taq-based polymerases) and an extension of 72 °C for 10 s, and a final extension of 8 min at 72 °C.

When the annealing temperature was ≥ 69 °C (as in the case of some of the species-specific primers shown in Supplemental Table 1), a two-step PCR protocol — with no annealing step — was used.

The resulting amplicons were gel-purified (NZYGelpure, NZYTech, Portugal) and cloned using CloneJET PCR Cloning Kit, Thermo Scientific). Plasmid DNA of ten clones from each positive sample was purified (NZYMiniprep, NZYTech, Portugal) and sequenced on an ABI 3730XL sequencing machine (GATC, Eurofins Genomics, Germany).

### Population Genetics Analyses

Based on the expression *P* = 1 − (1 − *p*) ^ *n*, where *P* is the probability of finding a variant at frequency *p* (in this case, *p* = 0.25) in a sample of size *n*, it was estimated that the sequencing of ten amplicons provided about a 95% likelihood of identifying any variant segregating at a frequency ≥ 25% in each isolate, which ensured enough power for the analysis of genetic variation.

It should be noted that although the ATCC 30254 strain was originally isolated from *A. mellifera*, for subsequent analyses, it was considered apart on the grounds that it came from an axenic culture. In the case of *GADPH,* two additional GenBank datasets encompassing the same region were included for comparison with ours (PopSets 1169070972 and 663527929).

The nucleotide diversity in each species was estimated at synonymous and nonsynonymous sites by means of the *π* [average number of nucleotide differences per site between two sequences; 34] and *θ*_*W*_ [number of segregating sites; 35] statistics, which were calculated applying the Jukes and Cantor correction [36]. Both measures are complementary, since *π* is sensitive to the frequency of polymorphisms while *θ*_*W*_ is not. These parameters as well as the Tajima’s *D* statistic [37] — a measure of the mutation frequency spectrum — were calculated with DnaSP v6 [38]. *D* values are negative when there is an excess of rare variants and positive when there is an excess of high-frequency mutations. The pooled value of *D* across *loci* was estimated manually using Tajima’s formulae [37]. The divergence (or genetic differentiation) between species was quantified as the mean number of synonymous (*K*_*s*_) and nonsynonymous (*K*_*a*_) substitutions per site, using the Jukes and Cantor correction, as implemented in DnaSP v6. Alignment gaps were excluded from all the analyses.

DnaSP v6 was also used to carry out the McDonald-Kreitman test [39], which was applied to each *locus* to assess if the levels of variability within and between species fitted the predictions of the neutral theory. Under this model, both should be correlated over time [40], and therefore the ratio of nonsynonymous to synonymous variation between species (*Dn/Ds*) should be equal to the ratio of nonsynonymous to synonymous variation within a species (*Pn/Ps*).

Median-joining haplotype networks [41] were constructed using PopART [42] (http://popart.otago.ac.nz/index.shtml) departing from nexus haplotype files generated with DnaSP v6. This type of networks includes nodes to represent inferred sequences by iteratively adding ‘median’ sequence vectors.

The analysis of molecular variance (AMOVA) among haplotypes was performed with Arlequin 3.5 [43]. The significance of the covariance components associated with different levels of genetic structure (within isolates, among isolates within host species and among host species) was tested using non-parametric permutation procedures [44].

The existence of recombination was initially tested with RecMin (http://www.stats.ox.ac.uk/~myers/RecMin.html), which provides two minimum numbers of recombination events: the statistic *Rm* of Hudson and Kaplan [45] based on the four-gamete test, and *Rh* [46], which reflects the number of recombination events required to explain the history of a sample under the assumption that each segregating site has mutated only once. However, recombination was only confirmed after performing a permutation-based analysis with LDhat [47], which uses the composite-likelihood method of Hudson [48], adapted to finite-sites models [47]. The maximum composite likelihood was calculated under random permutation of the physical position of the variants (1000 permutations) to test the hypothesis of no-recombination.

## Results

The protocol of PCR cloning and sequencing the amplicons obtained with broad-range primers revealed the presence of multiple trypanosomatid species in samples where *a priori* only one had been detected by direct sequencing (Table 1).

**Table 1:** Trypanosomatid species detected in *A. mellifera* and *B. terrestris* samples

Host	Isolate	Direct sequencing	Cloning sequencing
*A. mellifera*	ITS2 PR13-21	*L. passim*	*L. passim*
	ITS3 PR13-21	*L. passim*	*L. passim*
	PA11-831	*L. passim*	*L. passim*
	PA11-847	*L. passim*	*L. passim*
	PA11-853	*L. passim*	*L. passim*
	PA14-0015	*C. mellificae*	*L. passim*; *C. bombi*; *C. mellificae*
	PA14-0044	*C. mellificae*	*L. passim*; *C. bombi*; *C. mellificae*
*B. terrestris*	B14.213	*C. bombi*	*L. passim*; *C. bombi*
	14_349	*C. bombi*	*C. bombi*
	14_351	*C. bombi*	*L. passim*; *C. bombi*; *C. mellificae*
	14_373	*C. bombi*	*C. bombi*
	14_395	*C. bombi*	*L. passim*; *C. bombi*; *C. mellificae*

*L. passim* was the most prevalent trypanosomatid of the dataset, as it was identified in the seven field isolates of *A. mell*ifera and in three out of the five *B. terrestris*. In half of these samples, this pathogen co-occurred with, at least, a second species (Table 1).

In bumblebees, the most prevalent trypanosomatid species was *C. bombi*, which was detected in the five isolates studied.

### Population Genetics Analyses

#### Diversity Within Species

The first observation derived from these analyses was that the *loci* used in this study exhibited some heterogeneity in the levels of synonymous nucleotide variation (*π*_*S*_), *RPB1* being considerably more polymorphic than *GAPDH* or *TOPII* in *L. passim* and *C. mellificae* (Table 2 and Supplemental Tables 2, 3 and 4). This *locus* also showed slightly higher diversity in *C. bombi*, although it was not statistically different from that of *GAPDH*.

**Table 2:** Mean synonymous (*π*_*S*_) and nonsynonymous (*π*_*A*_) pairwise diversity per *locus* and species (± standard error, SE)

	Species	Mean *π*_*S*_ ± SE (%)	Mean *π*_*A*_ ± SE (%)
*GAPDH-A*	*C. bombi*	0.07 ± 0.03	0.03 ± 0.02
	*C. mellificae*	0.93 ± 0.17	0.07 ± 0.05
	*L. passim*	0.06 ± 0.03	0.06 ± 0.02
*GAPDH-B*	*C. bombi*	0.00 ± 0.00	0.00 ± 0.00
	*C. mellificae*	1.40 ± 0.29	0.30 ± 0.04
	*L. passim*	0.38 ± 0.06	0.76 ± 0.42
*RPB1*	*C. bombi*	0.13 ± 0.06	0.12 ± 0.03
	*C. mellificae*	4.21 ± 0.39	0.03 ± 0.02
	*L. passim*	0.96 ± 0.12	0.17 ± 0.02
*TOPII*	*C. bombi*	0.00 ± 0.00	0.05 ± 0.02
	*C. mellificae*	1.58 ± 0.41	0.06 ± 0.03
	*L. passim*	0.17 ± 0.07	0.16 ± 0.03

*GAPDH-A*: data from this work (ATCC 30254 excluded)

*GAPDH-B: C. bombi* (Popset 1169070972), *C. mellificae* (Popset 663527929 + ATCC 30254 from this work) and *L. passim* (Popset 663527929)

Overall, *C. mellificae* was far more diverse at synonymous positions (2.24 ± 1.00%) than *L. passim* (0.40 ± 0.28 %) or *C. bombi*, which showed extremely low levels of diversity (0.06 ± 0.04 %) (mean *π*_*S*_ ± SE estimated using data from Supplemental Tables 2, 3 and 4). These values remained nearly unchanged after weighting each *locus* by its number of synonymous sites, to avoid any bias caused by the different length of the genes: weighted mean *π*_*S*_ ± SE = 2.02 ± 0.91% for *C. mellificae*, 0.35 ± 0.30% for *L. passim* and 0.07 ± 0.04% for *C. bombi*.

The ATCC 30254 strain displayed a little more diversity across loci than the *C. mellificae* field samples, both using data from this study (weighted mean *π*_*S*_ ± SE = 3.01 ± 1.15%, estimated using data from Supplemental Tables 2, 3 and 4) and when considering sequences from other *C. mellificae* type isolates available in GenBank (*GAPDH*-B in Table 2). In the case of *L. passim*, the difference between the two groups was highly significant (0.38 ± 0.06 in cultured cell lines vs. 0.06 ± 0.03 in wild populations), whereas the field samples from the *C. bombi* Popset 1169070972 (*GAPDH*-B in Table 2) exhibited no variability.

Along with its higher rate of synonymous variation, *C. mellificae* displayed a greater tendency to present intermediate frequency mutations at the three *loci* (*D*_*S*_ pooled across *loci*= 1.67; data from Supplemental Tables 2, 3 and 4) than the other two species, which exhibited slight excesses of rare variants (*D*_*S*_ pooled across *loci*= −1.59 and −1.52 for *C. bombi* and *L. passim*, respectively; data from Supplemental Tables 2, 3 and 4). At any rate, *D*_*S*_ deviations only reached significant values at *GAPDH* in *C. mellificae* and *L. passim* (Supplemental Table 2).

At nonsynonymous sites, *C. mellificae* showed an important reduction in variability with respect to that at synonymous positions, both when the three *loci* were considered individually (Table 2) and when they were pooled together (weighted mean *π*_*A*_ ± SE = 0.05 ± 0.08% vs. weighted mean *π*_*S*_ ± SE = 2.02 ± 0.91%), in line with the values observed in the ATCC 30254 strain (weighted mean *π*_*A*_ ± SE = 0.11 ± 0.40% vs. weighted mean *π*_*S*_ ± SE = 3.01 ± 1.15%).

In contrast, *L. passim* and *C. bombi* displayed similar rates of polymorphism at both sites (weighted mean *π*_*A*_ ± SE = 0.12 ± 0.08% vs. weighted mean *π*_*S=*_ 0.35 ± 0.30% for *L. passim*, and weighted mean *π*_*A*_ ± SE =0.06 ± 0.09% vs. weighted mean *π*_*S*_ ± SE = 0.07 ± 0.04% for *C. bombi*, respectively).

The *GAPDH*-B datasets of *C. mellificae* and *L. passim* followed the same trends as those described above for each of these species, but with much higher rates of nonsynonymous diversity than those obtained in the natural populations analysed here (Table 2). Yet again, the *C. bombi* Popset 1169070972 showed no variability (*GAPDH*-B, Table 2).

In most cases, the frequency spectrum of replacement substitutions at individual genes did not deviate from neutral expectations (*D*_*A*_ values from Supplemental Tables 2, 3 and 4). However, when the mean *D*_*A*_ was calculated across *loci*, it displayed a significant excess of rare variants for the three species (*D*_*A*_ pooled across *loci* = −3.21, *P* < 0.001, for *C. bombi*; −2.62, *P* < 0.01, for *L. passim*; and −1.97, *P* < 0.05, for *C. mellificae*).

In terms of diversity (both at synonymous and nonsynonymous positions), there were no significant differences among trypanosomatid species isolated from different hosts, although this point will be addressed in detail in the ‘Population Structure’ section.

#### Divergence Between Species

The analysis of divergence between species revealed important differences among *loci*. *GAPDH* was the *locus* with least synonymous differentiation among species (*K*_*s*_), whereas *TOPII* exhibited saturation of synonymous mutations (*K*_*s*_ > 1) each time that *C. bombi* was involved in the comparisons (Table 3), which derived in larger mean *K*_*s*_ ± SE values across *loci* (0.85 ± 0.39 between *C. bombi* and *C. mellificae* or 0.79 ± 0.35 between *C. bombi* and *L. passim*) than those obtained from the comparison between *C. mellificae* and *L. passim* (0.50 ± 0.12).

**Table 3:** Pairwise nucleotide divergence, at synonymous (*K*_*s*_) and nonsynonymous sites (*K*_*a*_)

*Locus*	*C. bombi* vs. *C. mellificae*		
	*Ks*	*Ka*	*Ka/Ks*
*GAPDH*	0.24	0.01	0.04
*RPB1*	0.72	0.01	0.01
*TOPO*	1.59	0.06	0.04
*Locus*	*C. bombi* vs. *L. passim*		
	*Ks*	*Ka*	*Ka/Ks*
*GAPDH*	0.23	0.02	0.09
*RPB1*	0.71	0.01	0.01
*TOPO*	1.44	0.07	0.05
*Locus*	*L. passim* vs. *C. mellificae*		
	*Ks*	*Ka*	*Ka/Ks*
*GAPDH*	0.28	0.01	0.04
*RPB1*	0.54	0.00	0.01
*TOPO*	0.67	0.10	0.15

Again, *TOPII* was the gene accumulating most replacement variants between species (*K*_*a*_) although the average *K*_*a*_ ± SE values across *loci* were much more similar among them than those of *K*_*s*_ (*K*_*a*_ ± SE= 0.04 ± 0.03 between *C. mellificae* and *L. passim*, 0.03 ± 0.02 between *C. mellificae* and *C. bombi* or 0.03 ± 0.02 between *C. mellificae* and *L. passim*).

In all cases, the *K*_*a*_/ *K*_*s*_ ratios, which allow inferring the existence and direction of selective forces acting on a sequence, were lower than one, which is consistent with a predominant purifying selection (Table 3).

To further investigate the heterogeneity observed among genes, both in terms of polymorphism and divergence, we performed McDonald-Kreitman tests [39] to assess if the ratios of nonsynonymous and synonymous mutations within and between species fitted the predictions of the neutral model [40].

However, contrary to the expectations under neutrality (in which *Pn*/*Ps* =*Dn/Ds*; see ‘Materials and Methods’ section), the vast majority of the comparisons resulted in significantly higher *Pn/Ps* than *Dn/Ds* ratios (0.88 > 0.14 for pooled data between *C. bombi* and *C. mellificae*, 2.38 > 0.19 between *C. bombi* and*. L. passim* and 0.81 > 0.22 between *C. mellificae* and *L. passim*, respectively), indicating an excess of amino acid polymorphisms (*Pn*) as compared with fixations (*Dn*; Table 4).

**Table 4:** McDonald-Kreitman tests

*Cb* vs. *Cm*	*Locus*	Fixed		Polymorphic		*P*
		Syn	Non-syn	Syn	Non-syn	
	*GAPDH*	33	5	6	7	**
	*RPB1*	43	2	15	11	***
	*TOPII*	63	12	5	5	*
	Pooled	139	19	26	23	***
*Cb* vs. *Lp*	*Locus*	Fixed		Polymorphic		*P*
		Syn	Non-syn	Syn	Non-syn	
	*GAPDH*	33	9	8	14	**
	*RPB1*	47	2	4	15	***
	*TOPII*	60	15	4	9	***
	Pooled	140	26	16	38	***
*Cm* vs. *Lp*	*Locus*	Fixed		Polymorphic		*P*
		Syn	Non-syn	Syn	Non-syn	
	*GAPDH*	37	6	8	11	**
	*RPB1*	33	0	15	7	***
	*TOPII*	43	19	9	8	ns
	Pooled	113	25	32	26	***

*Cb*, *C. bombi*; *Cm*, *C. mellificae*; *Lp*, *L. passim*; *Fixed*, fixed mutations between species; *Ds and Dn*, number of synonymous and nonsynonymous fixed variants, respectively; *Polymorphic*, polymorphic changes within species; *Ps and Pn*, number of synonymous and nonsynonymous polymorphic variants, respectively; *P*, significance of the deviation from the neutral model calculated using the two-tailed Fisher’s exact test; *ns*, non-significant; *P < 0.05, **P < 0.01 and ***P < 0.001

When variants with frequencies below 5% were removed from the analyses, in order to increase the power to detect adaptive evolution [49], the differences between these ratios turned non-significant.

#### Population Structure

As previously mentioned, isolates obtained from honeybees and bumblebees showed non-significant differences in nucleotide diversity measured as *π* [34].

An alternative way to investigate if there was any kind of genetic structure among the haplotypes detected in these hosts was to estimate the significance and relative contribution of each of the covariance components to the total haplotypic variance (see AMOVA analysis in the ‘Materials and Methods’ section). This analysis showed that most of the variation resided within isolates (range 82.2–100%, Table 5), and this was valid for all species and markers except for *GAPDH* in *C. mellificae*, where the major contributor to the total variance was the variation among isolates of the same host (*P* < 0.001 in permutation tests). This component was the second contributor to the variance in *L. passim*, accounting for 14.1% of the total variance (average across the three *loci*). None of the three parasite species exhibited significant haplotype differentiation among hosts.

**Table 5:** Analysis of molecular variance (AMOVA)

Species	*Locus*	Source of variation	d.f.	SS	VC	% var	*P*
*C. bombi*	*GAPDH*	Among host species	1	0.1	0.0	−1.4	
		Among isolates within host species	5	0.7	0.0	0.3	
		Within isolates	63	8.1	0.1	101.1	
		Total	69	8.8	0.1		
	*RPB1*	Among host species	1	0.7	0.0	4.5	
		Among isolates within host species	5	1.5	0.0	1.9	
		Within isolates	63	16.1	0.3	93.6	*
		Total	69	18.3	0.3		
	*TOPII*	Among host species	1	0.2	0.0	3.8	
		Among isolates within host species	5	0.4	0.0	1.9	
		Within isolates	63	4.3	0.1	94.3	
		Total	69	4.9	0.1		
*C. mellificae†*	*GAPDH*	Among host species	1	2.5	−0.4	−45.1	
		Among isolates within host species	2	22.1	1.1	113.2	***
		Within isolates	36	10.9	0.3	32.0	***
		Total	39	35.4	0.9		
	*RPB1*	Among host species	1	4.2	0.1	5.5	
		Among isolates within host species	2	3.7	0.0	−0.8	
		Within isolates	36	72.4	2.0	95.3	
		Total	39	80.3	2.1		
	*TOPII*	Among host species	1	0.9	0.0	0.0	
		Among isolates within host species	2	1.7	0.0	1.2	
		Within isolates	36	27.4	0.8	98.9	
		Total	39	30.0	0.8		
*L. passim*	*GAPDH*	Among host species	1	0.3	0.0	−1.6	
		Among isolates within host species	8	3.2	0.0	11.3	***
		Within isolates	90	16.0	0.2	90.2	***
		Total	99	19.5	0.2		
	*RPB1*	Among host species	1	0.9	0.0	−4.4	
		Among isolates within host species	8	19.3	0.2	22.2	***
		Within isolates	90	58.7	0.7	82.2	***
		Total	99	78.9	0.8		
	*TOPII*	Among host species	1	0.6	0.0	0.1	
		Among isolates within host species	8	4.4	0.0	8.9	**
		Within isolates	90	24.9	0.3	91.0	**
		Total	99	29.8	0.3		

†ATCC 30254 excluded from the dataset

Negative values should be considered as zero, and values ≥100 as 100

*d*.*f*., degrees of freedom; *SS*, sum of squares; *VC*, variance components; % *var*, percentage of variation; *p*, probability of a random variance component value ≤ observed value in 3024 permutations; *p < 0.05, **p < 0.01 and ***p < 0.001

**Fig. 1:**
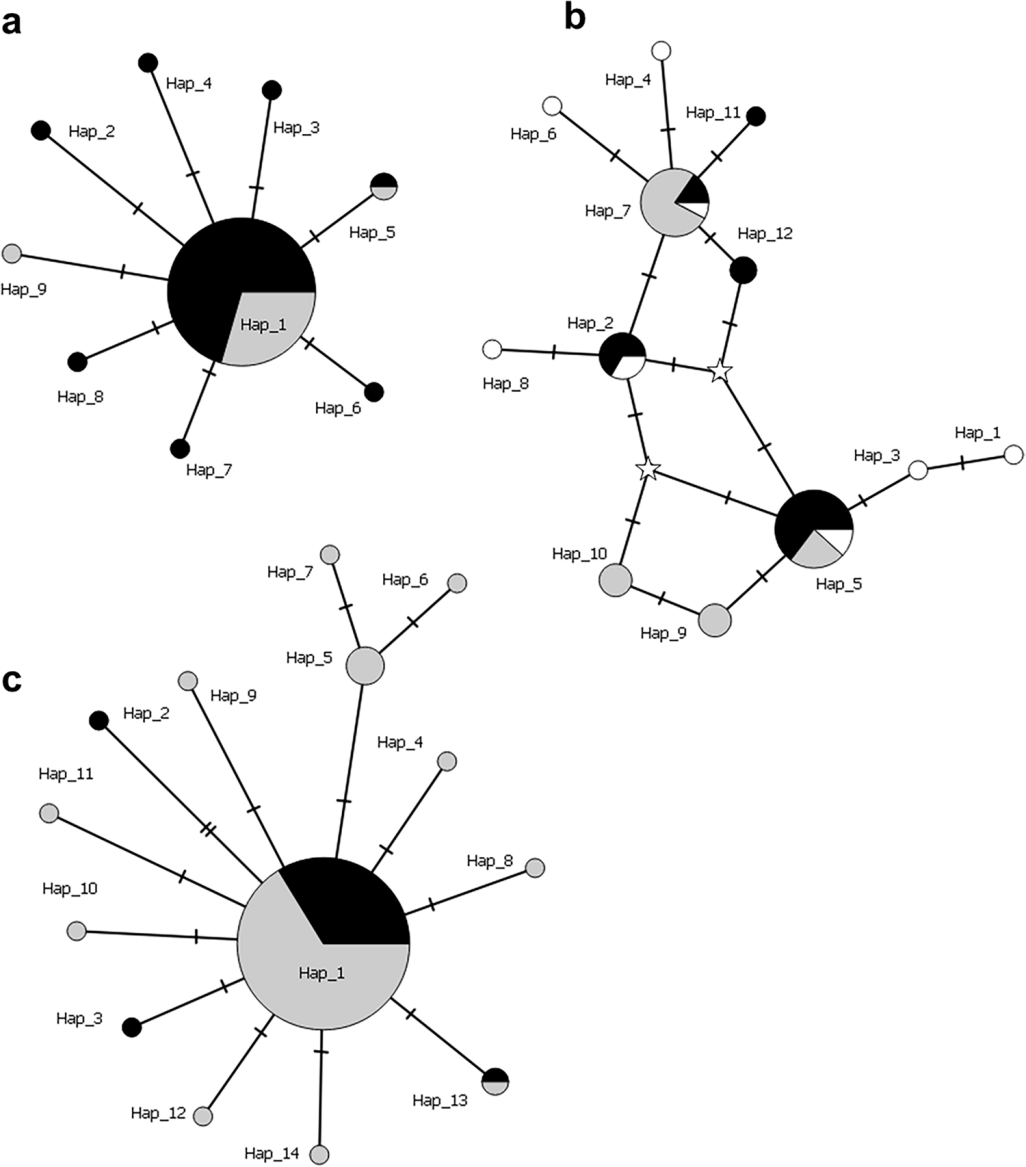
Median-joining haplotype network for *GAPDH*. Haplotypes are depicted by circles, the width being proportional to their frequencies. Black, grey and white circles/sections represent haplotypes obtained from *B. terrestris*, *A. mellifera* and the ATCC 30254 strain (this work), respectively. **a** *Crithidia bomb*i haplotypes; **b** *Crithidia mellificae* haplotypes; **c** *Lotmaria passim* haplotypes. Mutations are shown as hatch marks along edges and inferred haplotypes (‘median’ sequence vectors) are represented by stars

The graphical representation of the haplotypes revealed that they were organised in one or more (usually two) core haplotypes, present in both hosts at intermediate frequencies, from which derived low-frequency haplotypes that, in most cases, differed from the former by a single mutation (Fig. 1 and Supplemental Figs. 2 and 3). This became even more obvious when the sequences retrieved from GenBank were included in the networks, since their haplotypes were mostly represented by single sequences (data not shown).

#### Recombination

*L. passim* and C*. mellificae* displayed reticulated haplotype networks (Fig. 1 and Supplemental Figs. 2 and 3), which suggest the existence of recombination events in these species. Consistent with these patterns, *L. passim* showed evidences of potential recombination at *RPB1* (*Rm*= 2; *Rh*= 3), whereas *C. mellificae* exhibited them at all *loci* (*Rm*= 1 and *Rh*= 2 at *GAPDH*, *Rm*= 2 and *Rh*= 5 at *RPB1* and *Rm*= 1and *Rh*= 3 at *TOPII*). The statistical significance of these potential recombination events was evaluated with permutation tests (LDhat; [47]) and it proved significant (*P* < 0.01) for *RPB1* and *TOPII* in *C. mellificae*.

## Discussion

There is increasing evidence of the simultaneous presence of multiple trypanosomatids in bees [6, 14–16]. One of the reasons why these co-occurrences have not been reported until recently might be due to the methodology employed for their detection, which usually relies on the PCR amplification of DNA isolates followed by the direct sequencing of the PCR products [5, 9, 10, 50, 51]. However, as seen in this study, direct sequencing might overlook the presence of species with lower parasitic loads. This drawback can be solved by adding a cloning step prior to sequencing that although more expensive and time consuming is one of the best options for carrying out reliable population genetics analyses, not only because it allows the detection of additional species but also because it enables to uncover the presence of low-frequency variants within a species [8, 15, 25]. As an example, we can mention the Popset 1169070972 that we intended to use for comparison with our *C. bombi* sequences and that, despite of being a fairly large dataset, showed no variation likely due to direct sequencing (V. Vavilova personal communication).

Thus, in the current study, we cloned and sequenced 10 amplicons of *GAPDH*, one of the genes most frequently used for the molecular identification of trypanosomatids [8, 15, 25, 52], and of two other single-locus nuclear markers (*RPB1* and *TOPII*) for which there were no previous population data.

One of the findings resulting from their analysis, and that contrasts with previous reports based on microsatellite data [21, 53], was the low variability detected in *C. bombi*, both at synonymous and non-synonymous sites. The discrepancy between both types of variation data probably relies on the higher mutation rate shown by microsatellites with respect to single-copy coding regions of the genome [54]. Besides, microsatellites are often subject to homoplasy (identical sequences and/or amplicon sizes resulting from independent evolutionary paths) that, together with other factors [reviewed by 24], may confound the population genetic inference. Although the analysis of a few genes does not necessarily reflect the general patterns of variation of a species, the consistency of the trends detected across the three *loci* seems to suggest that *C. bombi* is little polymorphic, at least in these isolates. The reduced nucleotide variation together with a negative Tajima’s *D*_*S*_ shown by *C. bombi* is consistent with a recent population expansion [55] that could be related with the radiation of bumblebees from Central Asia [56]. In agreement with this, Central European samples of *C. bombi* were found to be less diverse than those from Alaska, which are closer to their basal populations [27]. Besides, Gerasimov et al. [27] found lower variability in the mitochondrial DNA of *C. bombi* than in its nuclear counterpart, which is largely compatible with a bottleneck scenario: the population undergoes a drastic decrease in size that results in a reduction in the levels of variation that, at first, is more evident in the mitochondrial DNA because its smaller effective population size makes it more responsive to demographic changes than the nuclear one [57].

To the best of our knowledge, the only other population genetic study of genetic variation in *L. passim* is that of Cepero et al. [25], who reported the first genetic description of this trypanosomatid prior to its formal naming and characterization by Schwarz et al. [8]. The re-analysis of the section of Cepero’s sequences that overlapped with ours (KJ704252.1-KJ704272.1; positions 50 to 499) revealed significantly higher diversity than that obtained in the current study both at synonymous (*π*_*S*_ ± SE= 0.32 ± 0.12 vs. 0.06 ± 0.03) and nonsynonymous sites (*π*_*A*_ ± SE= 0.50 ± 0.30 vs. 0.06 ± 0.02), in line with the results obtained for the *L. passim* sequences obtained from axenic cultures (Popset 663527929; *π*_*S*_ ± SE= 0.38 ± 0.06 and *π*_*A*_ ± SE= 0.76 ± 0.42), which suggest large differences in the levels of polymorphism across samples (especially at nonsynonymous positions, as proved by the large SE values). Both groups of field isolates (Cepero’s and those obtained in this work) exhibited significantly negative Tajima’s *D* at synonymous sites (− 1.87 and − 1.91, respectively; *P* < 0.05). The significance of this statistic at *GAPDH*, together with the excess of rare neutral variants observed in our dataset of *L. passim* (*D*_*S*_ pooled across *loci*= −1.52), seems to indicate that the natural populations of this species have been subject to a recent demographic expansion which could hypothetically be associated with its spread to new geographical areas.

On the other hand, *C. mellificae* proved to be considerably more diverse at synonymous positions than *C. bombi* or *L. passim*, which suggests that it may have a larger population size than any of these two species. It also exhibited a greater tendency to present intermediate frequency mutations than the former ones (represented by positive Tajima’s *D* values). This can indicate either the existence of balancing selection, population structuring or a sudden population contraction; the first possibility was discarded on the basis that balancing selection usually affects very specific targets, whereas in this case, the three *loci* showed a similar pattern; the results of the analysis of molecular variance also permitted to disregard the existence of population subdivision (most of the diversity resided within isolates), so the most probable explanation for the presence of this slight excess of intermediate frequency alleles is the existence of a reduction in population size (following an incomplete bottleneck, low-frequency mutations are lost more rapidly than common ones, deriving in transient positive Tajima’s *D* values) [55, 57].

Independently of the demographic trends shown by each of these trypanosomatids, the most relevant pattern for the three species is that they are subject to the action of purifying selection on nonsynonymous variants, which is a process that removes deleterious mutations (usually causing amino acid changes) from the populations. Its effect was detected even in *C. bombi*, where the efficiency of purifying selection was likely to be lower due to its recent bottleneck and concomitant reduction in effective population size [58].

The existence of such selective constraints on the three species comes from different evidences: (i) the frequency spectrum of replacement changes, represented by significantly negative *D*_*A*_ values, indicated that a large part of these mutations were segregating at low frequencies [37, 54] and (ii) *Ka/Ks* ratios lower than one suggested that mutations modifying a protein were less likely to get fixed between two species than synonymous substitutions [59].

Both findings were clearly reflected in the results of the McDonald-Kreitman test, in which the removal of polymorphisms with a frequency below 5% confirmed that the excess of replacement polymorphisms (*Pn*) observed prior to their exclusion was mostly due to the presence of slightly deleterious mutations, which usually segregate at low frequencies and rarely become fixed in the populations [49, 60]. This surplus of rare mutations could also be inferred from the haplotype networks, which in most cases exhibited one or two intermediate-frequency haplotypes connected to other closely related haplotypes that often represented singleton mutations, whose abundance is also dependent on the recombination rate [61]. However, it should be taken into account that pooling potentially different allopatric samples into single specific groups may interfere with the analysis of this parameter through the risk of a Wahlund effect [62].

The apparent absence of recombination events observed in this study for *C. bombi* would lead to a reduction in the effectiveness of selection [63] that would contradict the results of the MacDonald-Kreitman test; this suggests that, although not detected here, there must be a certain genetic exchange that helps to maintain the integrity of the genome. Consistent with this, *C. bombi*, as well as other trypanosomatids, showed evidence of Mendelian segregation in hybridization experiments [64, 65], a finding that was further supported by the presence of meiosis-specific genes in their genomes [66–68]. In this study, we also found signs of recombination in *L. passim* and *C. mellificae*, especially in the latter, where the evidence for this process was highly significant and in good agreement with the high levels of synonymous diversity and the efficiency of purifying selection (*π*_*A*_ << *π*_*S*_) observed in this species.

Another characteristic common to the three species is that most of the variation resided within isolates, which means that the haplotypes detected for the different trypanosomatids showed no structuring according to the host, suggesting that direct and indirect interactions between pollinators enable the dispersal of the most common haplotypes of each species, which are present both in honeybees and bumblebees (as depicted in the haplotype networks from the previous section). Also, the fact that parasites show similar levels of diversity in both hosts contradicts the assumption that those present in their main host should have a larger population size than those carried by incidental vectors, which, again, seems to indicate that most of the parasite variability is effectively spread and maintained into the environment by the former. Finally, it should be stressed that although some population structuring was detected for *C. mellificae* and *L. passim* within hosts, the limited sample size prevents to draw any firm conclusion about the possible existence of genetic subdivisions within these species.

Altogether, this study represents a first approximation to the population genetics of these highly prevalent pathogens, which could serve as a basis to carry out further research involving more samples and genes.

## Supplementary Information

ESM 1 (PDF 244 kb)

ESM 2 (PDF 59 kb)

ESM 3 (PDF 42 kb)

ESM 4 (XLSX 10 kb)

ESM 5 (XLSX 15 kb)

ESM 6 (XLSX 15 kb)

ESM 7 (XLSX 15 kb)
