# Supplementary material for: Bee Trypanosomatids: First Steps in the Analysis of the Genetic Variation and Population Structure of Lotmaria passim, Crithidia bombi and Crithidia mellificae
Source: Microb Ecol. 2021 Oct 5;84(3):856–67. doi: 10.1007/s00248-021-01882-w (PMC9622509; doi:10.1007/s00248-021-01882-w)
Supplement: Supplementary file 1 — (PDF 244 kb) [file 248_2021_1882_MOESM1_ESM.pdf]

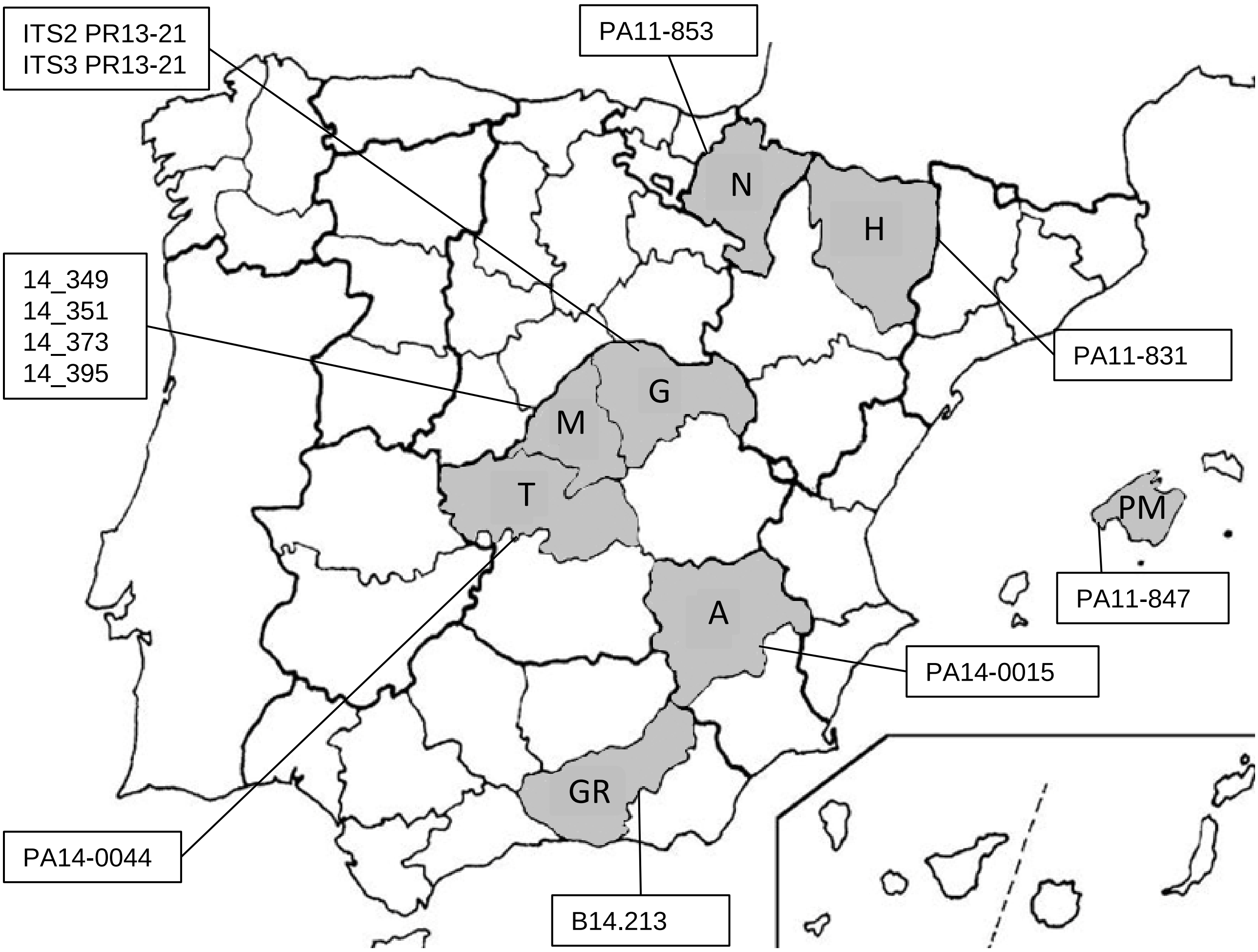

Supplemental Figure 1 Trypanosomatid-positive samples selected for the diversity study. *B. terrestris* specimens are named B14. or 14_ followed by a number; the remaining ones correspond to *A. mellifera*. A: Albacete; G: Guadalajara; GR: Granada; H: Huesca; M: Madrid; N: Navarra; PM: Palma de Mallorca; T: Toledo
