# Supplementary material for: Bee Trypanosomatids: First Steps in the Analysis of the Genetic Variation and Population Structure of Lotmaria passim, Crithidia bombi and Crithidia mellificae
Source: Microb Ecol. 2021 Oct 5;84(3):856–67. doi: 10.1007/s00248-021-01882-w (PMC9622509; doi:10.1007/s00248-021-01882-w)
Supplement: Supplementary file 3 — (PDF 42 kb) [file 248_2021_1882_MOESM3_ESM.pdf]

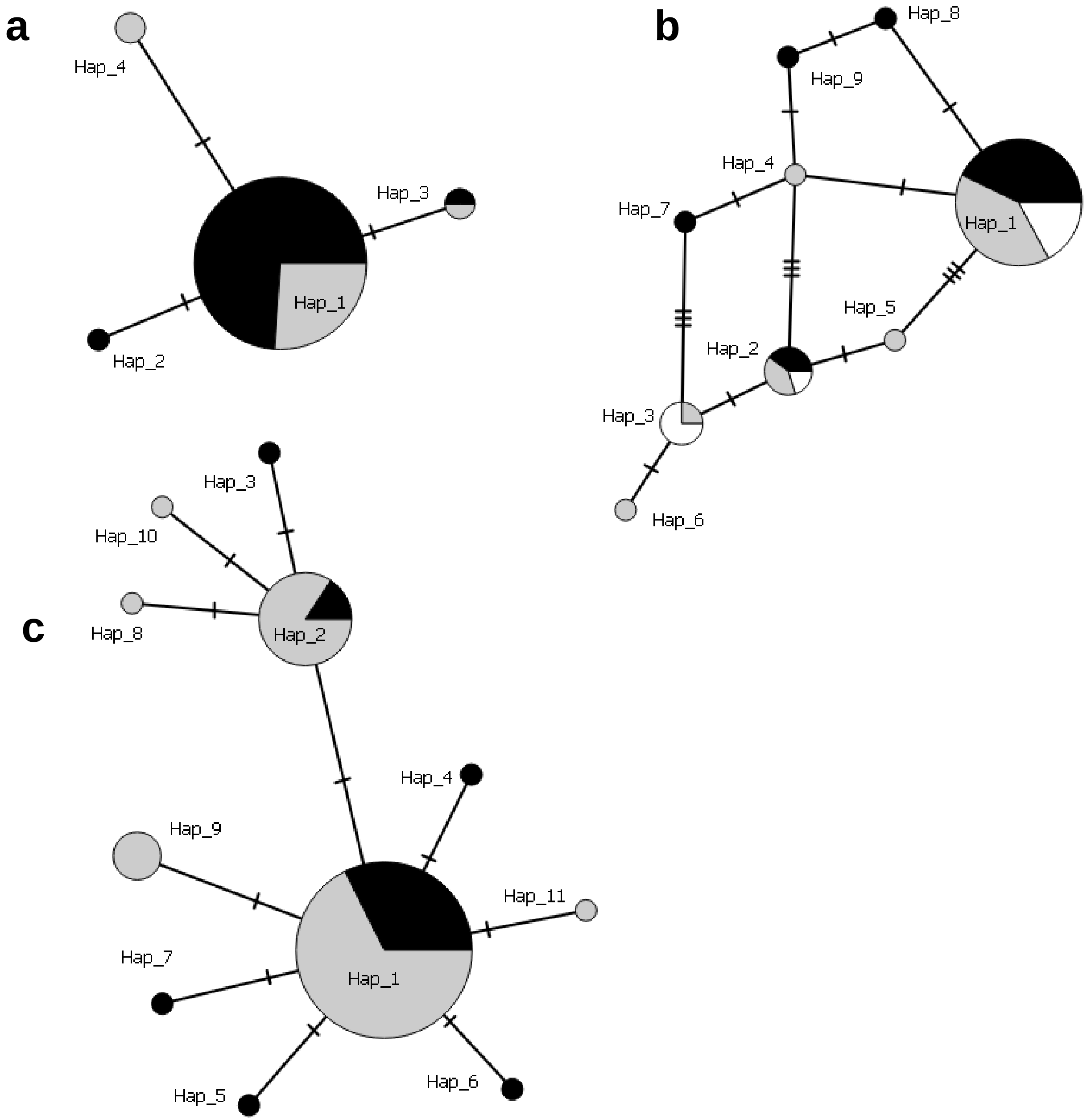

Supplemental Figure 3. Median-joining haplotype network for *TOPII*. Haplotypes are depicted by circles, the width being proportional to their frequencies. Black, grey and white circles/ sections represent haplotypes obtained from *B. terrestris*, *A. mellifera* and the ATCC® 30254™ strain, respectively. a: *Crithidia bombi* haplotypes; b: *Crithidia mellificae* haplotypes; c: *Lotmaria passim* haplotypes. Mutations are shown as hatch marks along edges.
